# A national survey of Brazilian endocrinologists’ practices in educating patients with adrenal insufficiency on stress-induced glucocorticoid adjustments

**DOI:** 10.20945/2359-4292-2025-0098

**Published:** 2025-08-18

**Authors:** Leonardo Vieira Neto, Aline Barbosa Moraes, Giselle Fernandes Taboada

**Affiliations:** 1 Hospital Universitário Clementino Fraga Filho, Departamento de Clínica Médica, Universidade Federal do Rio de Janeiro, Rio de Janeiro, RJ, Brasil; 2 Hospital Universitário Antônio Pedro, Departamento de Medicina Clínica, Universidade Federal Fluminense, Niterói, RJ, Brasil; 3 Disciplina de Clínica Médica, Universidade Estácio de Sá/IDOMED, Rio de Janeiro, RJ, Brasil

**Keywords:** Adrenal insufficiency, Adrenal crisis, Stress-induced glucocorticoid adjustments, Educational practices in adrenal insufficiency, Brazilian national survey

## Abstract

**Objective:**

To investigate the practices of Brazilian endocrinologists in educating patients with adrenal insufficiency about stress-induced glucocorticoid adjustments.

**Methods:**

This was a cross-sectional online survey carried out with 280 endocrinologists across Brazil. The survey included demographic questions and ten clinical vignettes assessing knowledge of appropriate glucocorticoid adjustments during various stressful situations. All participants provided informed consent, and the study protocol was approved by the local Ethics Committee. Statistical analysis compared responses based on physician demographics and practice settings.

**Results:**

The mean percentage of correct answers was 63.3%. A significant proportion of respondents (41.1%) incorrectly believed that patients should not self-administer intramuscular hydrocortisone during an adrenal crisis. Older physicians tended to provide more conservative (and potentially harmful) glucocorticoid dosing recommendations in certain scenarios. Physicians working in both outpatient and hospital settings demonstrated better knowledge of patient education and emergency glucocorticoid administration.

**Conclusion:**

The results of this study revealed moderate adherence to guidelines among Brazilian endocrinologists regarding adrenal insufficiency management and patient education. There is a need for improved education on glucocorticoid self-administration and targeted interventions to address knowledge gaps across different clinical scenarios. Further research is needed to evaluate the impact of these findings on patient outcomes and develop strategies to optimize the management of adrenal insufficiency in Brazil.

## INTRODUCTION

An adrenal crisis is a life-threatening condition that requires immediate medical attention due to its rapid onset and potentially fatal consequences. It is characterized by acute adrenal insufficiency, in which the production of glucocorticoids and/or mineralocorticoids is insufficient to meet the body’s physiological demands during periods of stress or illness. This condition primarily affects individuals with primary or secondary adrenal insufficiency, often resulting from autoimmune destruction or surgical removal of the adrenal glands, glucocorticoid withdrawal, or pituitary disorders that affect the secretion of adrenocorticotropic hormone (^1-3^).

The hallmark signs of an adrenal crisis include severe hypotension, electrolyte imbalances (such as hyponatremia and hyperkalemia), and metabolic abnormalities (*e.g.*, hypoglycemia). Prompt recognition and intervention are crucial to prevent the rapid progression to shock, multiple organ failure, and death (^3^). Despite its potentially devastating consequences, adrenal crisis remains under-recognized and inadequately managed in clinical practice. Epidemiological studies on adrenal crises have found an incidence of approximately five and ten adrenal crises per one hundred patient-years among patients with adrenal insufficiency treated with a standard replacement dose of glucocorticoid (^3-7^).

Recent epidemiological studies highlight the significant burden of morbidity and mortality associated with adrenal insufficiency and its acute exacerbations. This underscores the critical need for improved patient education and physician guidance. Effective patient education empowers individuals to recognize prodromal symptoms of adrenal crisis, such as fatigue, nausea, and dizziness, and to understand the importance of timely glucocorticoid supplementation during stressful situations. Furthermore, attending physicians play a pivotal role in ensuring patients receive adequate education, regular monitoring, and personalized management plans tailored to their adrenal function status (^8-10^).

This study aimed to investigate the practices of Brazilian endocrinologists in educating patients with adrenal insufficiency about stress-induced glucocorticoid adjustments. Understanding current educational practices may help design continuing medical education programs, contributing to highlighting strategies to optimize patient care and minimize the risk of adrenal crisis in clinical practice.

## METHODS

This was a cross-sectional, observational study using data obtained from an online survey. All participants provided informed consent, and the study protocol was approved by the local Ethics Committee in June 2024 (CAAE 74855523.4.3001.5257).

### Participants

Endocrinologists throughout Brazil were invited to participate in the study.

The calculation of the sample size utilized confidence intervals for population proportions. Assuming a population of 5,000 endocrinologists in Brazil, as reported by the *Sociedade Brasileira de Endocrinologia e Metabologia*, and considering a margin of error of 5% and a confidence level of 90%, the estimated sample size was 257 respondents.

### Methods

The study utilized an online survey platform developed on Google Forms. The survey collected sociodemographic data, including respondents’ age, time since graduation and specialization, and practice setting (whether in the public and/or private sector). It then presented eight hypothetical clinical cases encompassing common scenarios requiring glucocorticoid dose adjustments in patients with adrenal insufficiency. The study included questions relevant to the management of both primary and secondary adrenal insufficiency. All recommended glucocorticoid dose adjustments to prevent an adrenal crisis were based on the current Endocrine Society guideline (^2^). The applied questionnaire consisted of two general questions and eight clinical vignettes, described below. Correct answers are presented in uppercase letters. Incorrect answers were categorized into two classes: those that could pose harm to the patient or risk of adrenal crisis (highlighted in bold) and those that would not cause harm to the patient or risk of death (highlighted in italics):

**Question #1:** Education/guidance on adjusting the prednisone dose during stressful situations should be provided: A) *At the first consultation and at subsequent consultations as deemed necessary by the physician*; B) *When the patient has doubts or demands in the face of stressful situations*; C) AT EVERY CONSULTATION, REGARDLESS OF THE PATIENT’S DEMAND OR DOUBTS; and D) **It should be discouraged due to the risk of abuse and the consequent development of complications associated with glucocorticoid excess.**

**Question #2:** Regarding the self-administration of glucocorticoid (hydrocortisone) via the intramuscular route in situations of stress for the prevention or treatment of an adrenal crisis: A) **The layperson (patient, family member, and/or caregiver) should not administer intramuscular hydrocortisone due to the risk of needle-stick injuries;** B) **Injectable medications should only be administered in a hospital setting by trained individuals due to the risk of complications such as hematoma and abscess formation;** C) **It should be discouraged due to the risk of abuse and consequent development of complications associated with glucocorticoid excess; and** D) IT SHOULD BE ACTIVELY ENCOURAGED BY THE ATTENDING PHYSICIAN, AND PATIENTS SHOULD BE EDUCATED ON ITS ADMINISTRATION.

**Question #3:** A patient will undergo a cesarean section. What guidance should be provided regarding the procedure? A) *Double the dose of prednisone on the day of delivery*; B) *Triple the dose of prednisone on the day of delivery*; C) ADMINISTER 100 MG OF INTRAVENOUS HYDROCORTISONE AT ANESTHESIA INDUCTION FOLLOWED BY 100 MG OF INTRAVENOUS HYDROCORTISONE EVERY 6 HOURS; and D) **Maintain the dose due to the risk of maternal-fetal complications from higher doses of administered glucocorticoid.**

**Question #4:** A patient will undergo a vaginal delivery. What guidance should be provided regarding the procedure? A) *Double the dose of prednisone on the day of delivery*; B) *Triple the dose of prednisone on the day of delivery*; C) ADMINISTER 100 MG OF INTRAVENOUS HYDROCORTISONE UNTIL THE ONSET OF THE ACTIVE PHASE OF LABOR, FOLLOWED BY 100 MG OF INTRAVENOUS HYDROCORTISONE EVERY 6 HOURS; and D) **Maintain the dose due to the risk of maternal-fetal complications from higher doses of glucocorticoid administration.**

**Question #5:** A patient will undergo dental calculus removal. What guidance should be provided regarding the procedure? A) *Double the dose of prednisone on the day of the procedure*; B) *Triple the dose of prednisone on the day of the procedure*; C) *Administer 100 mg of intravenous or intramuscular hydrocortisone at the beginning of the procedure*; and D) TAKE AN ADDITIONAL DOSE OF GLUCOCORTICOID (*E.G.*, 5 MG OF PREDNISONE) 60 MINUTES BEFORE THE PROCEDURE.

**Question #6:** A patient has vomiting and watery diarrhea due to gastroenteritis and is afebrile. She reported having vomited about 5 minutes after taking prednisone. What is the appropriate guidance regarding glucocorticoid administration? A) *Double the dose of prednisone until the condition resolves*; B) *Triple the dose of prednisone until the condition resolves*; C) ADMINISTER 100 MG OF INTRAMUSCULAR HYDROCORTISONE AND REPEAT IT AFTER 6-12 HOURS UNTIL RECOVERY; and D) **Maintain the prednisone dose due to the risk of immunosuppression in the presence of an infection and prescribe symptomatic treatment for the gastroenteritis.**

**Question #7:** A patient has a flu-like syndrome accompanied by fever, with a maximum axillary temperature of 38.2°C, and oxygen saturation ranging between 96% and 98%. What is the appropriate guidance regarding glucocorticoid administration? A) DOUBLE THE DOSE OF PREDNISONE UNTIL THE CONDITION RESOLVES; B) *Triple the dose of prednisone until the condition resolves*; C) *Administer 100 mg of intramuscular hydrocortisone and repeat it after 6-12 hours until recovery*; and D) **Maintain the prednisone dose due to the risk of immunosuppression in the presence of an infection and prescribe symptomatic treatment for the upper respiratory tract infection.**

**Question #8:** A patient has a flu-like syndrome accompanied by a maximum axillary temperature of 37.0°C and oxygen saturation ranging between 96% and 98%. What is the appropriate guidance regarding glucocorticoid administration? A) *Double the dose of prednisone until the condition resolves*; B) *Triple the dose of prednisone until the condition resolves*; C) *Administer 100 mg of intramuscular hydrocortisone and repeat it after 6-12 hours until recovery*; and D) MAINTAIN THE PREDNISONE DOSE.

**Question #9:** A patient develops epilepsy after a stroke and requires carbamazepine. Regarding the prednisone dose, it is correct to state that: A) *There is no need to adjust the prednisone dose*; B) THE PREDNISONE DOSE SHOULD BE INCREASED DUE TO ITS REDUCED SERUM LEVEL CAUSED BY THE CONCOMITANT ADMINISTRATION OF THE ANTICONVULSANT; C) **The prednisone dose should be reduced due to its increased serum level caused by the concomitant administration of the anticonvulsant; and** D) **The prednisone dose should be reduced due to the risk of new thromboembolic events induced by the glucocorticoid.**

**Question #10:** A patient will perform strenuous physical exercise. What is the appropriate guidance regarding glucocorticoid administration? A) *There is no need to adjust the prednisone dose for any intensity of physical activity*; B) ADMINISTER 2.5 MG OF PREDNISONE 30-60 MINUTES BEFORE PHYSICAL ACTIVITY; C) *Triple the prednisone dose on the day of the physical activity*; and D) *Administer 100 mg of hydrocortisone IM immediately before starting the physical activity*.

The survey was disseminated through multiple messaging platform groups (WhatsApp) composed of endocrinologists across Brazil. The researchers, who are members of these groups, requested further sharing of the survey with relevant endocrinology communities. The invitation to participate in the survey included a message outlining the study’s purpose and a link to the informed consent form and online questionnaire. To maximize participation, the invitation was sent on multiple occasions with biweekly intervals over an 8-week period. Survey responses were downloaded in Excel for subsequent analysis.

### Statistical analysis

The statistical analyses were performed using Statistical Package for Social Sciences (SPSS), version 23.0 for MacOS (SPSS Inc., Chicago, IL, USA). In the descriptive analysis, categorical variables were expressed as frequency and percentage, while numerical variables were expressed as mean ± standard deviation. Student’s *t* test was performed to compare numerical variables between two groups. Analysis of variance (Anova) was used to compare numerical variables among three groups, and the Tukey *post-hoc* test was applied to identify significant differences between pairs of groups, accordingly. The Chi-squared test or Fisher’s exact test was applied to compare categorical variables, as appropriate. Correlations between numerical variables were analyzed using the Pearson test. A p-value < 0.05 was considered significant.

## RESULTS

### Participant demographics

**Figure 1:**
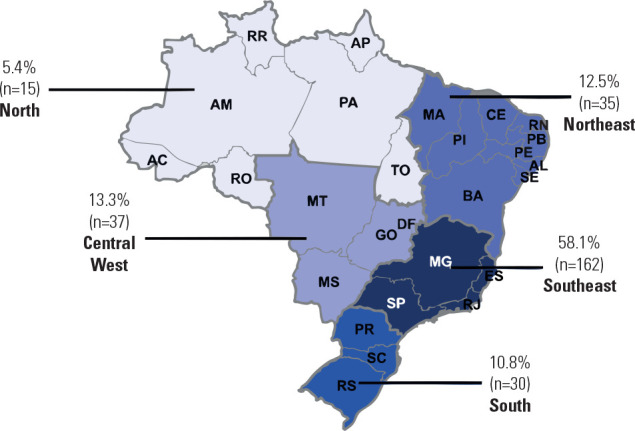
Regional distribution of study participants.

A total of 280 physicians voluntarily completed the online questionnaire. The study population had the following mean values: age 43.89 ± 10.5 years; years since graduation 19.42 ± 10.8 years; and years of specialization 14.53 ± 11.3 years. The participants represented 24 of Brazil’s 27 states (**Figure 1**), with the following regional distribution, presented as numbers (percentages): North, 15 (5.4%); Northeast, 35 (12.5%); Central-West, 37 (13.3%); Southeast, 162 (58.1%); and South, 30 (10.8%).

The majority of respondents (n = 212; 75.7%) had practiced for more than 5 years. Regarding the practice setting, 81 participants (29.0%) worked exclusively in the private sector, 26 (9.3%) exclusively in the public sector, and 172 (61.7%) in both. Most participants (n = 195; 69.9%) worked solely in outpatient clinics/offices, while 84 (30.1%) worked in both outpatient settings and hospitals (including wards, emergency rooms, or intensive care units).

### Questionnaire performance

**Figure 2:**
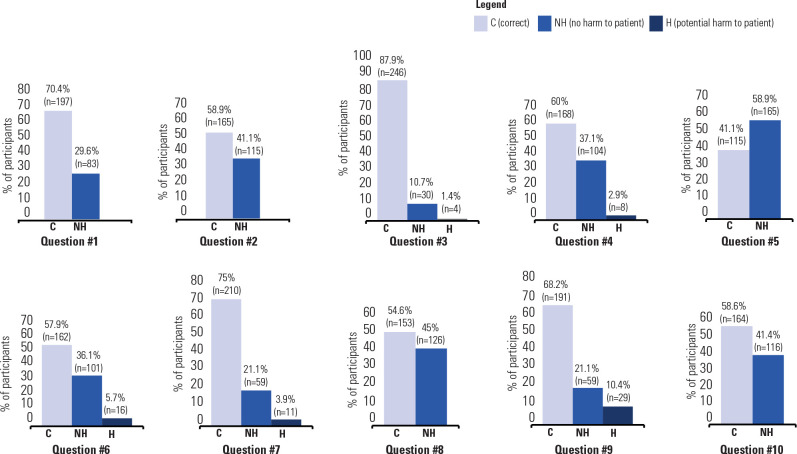
Percentage of answers to each question categorized as correct (C), incorrect but not representing harm (NH), incorrect with potencial harm to the patient (H).

The mean percentage of correct answers across all questions was 63.3%. Question #3 had the highest percentage of correct answers (87.9%). Conversely, Question #2 had the highest percentage of responses posing potential harm to the patient or risk of adrenal crisis (41.1%). A detailed breakdown of responses for each question, categorized as representing potential harm/risk, not representing harm/risk, and correct answers, is presented in **Figure 2** and **Table 1**.

**Table 1:** Age, time since graduation, and time since specialization across answer types

	Age (years)	p-value	Time since graduation (years)	p-value	Time since specialization (years)	p-value
Question #1						
Correct	43.2 ± 10.1	ns	18.6 ± 10.3	ns	13.7 ± 10.8	ns
No harm	45.6 ± 11.5		21.3 ± 11.8		16.4 ± 12.5	
Question #2						
Correct	44.0 ± 10.2	ns	19.5 ± 10.4	ns	14.6 ± 10.8	ns
Potential harm	43.8 ± 11.1		19.4 ± 11.5		14.4 ± 12.1	
Question #3						
Correct	43.6 ± 10.0	0.008^*^	19.1 ± 10.2	0.01^*^	14.2 ± 10.9	0.01^*^
No harm	44.1 ± 13.4	0.01^*^	19.9 ± 13.7	0.03^*^	14.7 ± 13.2	0.02^*^
Potential harm	59.5 ± 12.4		34.2 ± 11.7		30.7 ± 11.6	
Question #4						
Correct	45.1 ± 10.1		20.5 ± 10.3		15.9 ± 11.0	
No harm	42.1 ± 10.7	ns	17.7 ± 11.2	ns	12.4 ± 11.4	ns
Potential harm	42.7 ± 15.5		17.9 ± 14.6		13.5 ± 15.4	
Question #5						
Correct	45.2 ± 10.8		20.3 ± 11.1		15.5 ± 11.7	
No harm	42.9 ± 10.4	ns	18.8 ± 10.7	ns	13.9 ± 11.2	ns
Potential harm	42.5 ± 2.1		18.5 ± 2.1		12.0 ± 4.2	
Question #6						
Correct	45.8 ± 10.6	< 0.001†	21.5 ± 10.9	< 0.001†	16.7 ± 11.6	< 0.001†
No harm	39.7 ± 8.2		14.9 ± 8.1		10.1 ± 8.8	
Potential harm	50.8 ± 15.0	< 0.001†	26.5 ± 15.2	< 0.001†	21.3 ± 14.3	0.01†
Question #7						
Correct	43.7 ± 10.6		19.2 ± 10.7		14.4 ± 11.4	
No harm	43.5 ± 9.7	ns	18.9 ± 10.4	ns	13.9 ± 10.7	ns
Potential harm	50.4 ± 13.0		25.8 ± 12.8		20.0 ± 13.1	
Question #8						
Correct	45.0 ± 10.9	0.03‡	20.4 ± 11.0	ns	15.7 ± 11.5	0.03‡
No harm	42.3 ± 9.9		18.0 ± 10.3		12.8 ± 10.7	
Question #9						
Correct	43.3 ± 10.0		18.8 ± 10.3		13.9 ± 10.8	
No harm	44.9 ± 11.7	ns	20.7 ± 11.9	ns	15.6 ± 12.6	ns
Potential harm	44.5 ± 10.5		20.0 ± 10.4		15.7 ± 11.0	
Question #10						
Correct	43.1 ± 10.2	ns	18.5 ± 10.4	ns	13.4 ± 10.7	ns
No harm	45.0 ± 11.0		20.7 ± 11.2		16.1 ± 12.1	

Data are presented as mean ± standard deviation.

Answers were categorized as “correct”, “not representing harm/risk (no harm),” and “representing potential harm/risk”.

* p-value for comparison with responses with potential harm; † p-value for comparison with responses not representing harm; ‡ p-value for comparison between correct and response not representing harm.

ns: non-significant.

One participant did not answer questions #6, #8 and #9.

### Comparison of numerical variables

No significant differences were observed in age, years since graduation, or years since specialization based on medical practice setting, medical practice sector, Brazilian region, or responses to Questions #1, #2, #4, #5, #7, #9, and #10.

Significant differences in age, years since graduation, and years since specialization were found for responses to Questions #3, #6, and #8. In Question #3, mean age was significantly higher among those whose responses posed potential harm/risk of adrenal crisis (59.5 ± 12.4 years) compared with those whose responses did not pose harm/risk (44.1 ± 13.4 years; p = 0.001) and those with correct answer (43.6 ± 10.0 years; p = 0.008). Consequently, mean years since graduation and specialization were also significantly higher in the group providing the response that posed potential harm/risk (**Table 1**). In Question #6, participants whose responses posed potential harm/risk (50.8 ± 15.0 years) and those with the correct answer (45.8 ± 10.6 years) were significantly older than those whose responses did not pose harm/risk (39.7 ± 8.2 years; p < 0.001 for both comparisons). The distribution of responses according to mean years since graduation and specialization followed the same pattern (**Table 1**). In Question #8, the respondents’ mean age was significantly higher for those marking the correct answer (45.0 ± 10.9 years) compared with those choosing the response posing no harm/risk (42.3 ± 9.9 years; p = 0.03). The distribution of responses according to mean years since specialization followed the same pattern (**Table 1**).

### Comparison of frequencies

#### Years since graduation

No significant differences were found in the frequencies of responses for Questions #1, #2, #3, #5, #7, #9, and #10 based on years since graduation (≤ 5 years versus > 5 years). However, respondents with > 5 years since graduation, compared with those with ≤ 5 years, had significantly higher frequencies of correct answers for Questions #4 (64.6% *versus* 45.6%, respectively; p = 0.01), #6 (64.0% *versus* 39.7%, respectively; p = 0.001), and #8 (58.3% *versus* 44.1%, respectively; p = 0.04) (**Table 2**).

**Table 2:** Years since graduation, medical practice setting, and medical practice sector across answer types

	Years since graduation (%)				Medical practice setting (%)				Medical practice sector (%)			
	≤ 5	> 5	p-value		Outpatient clinic/office	Outpatient clinic/office and hospital	p-value		Private	Public	Both	p-value
Question #1												
Correct	77.9	67.9	ns		66.2	81.0	0.01		74.1	76.9	68.0	ns
No harm	22.1	32.1			33.8	19.0			25.9	23.1	32.0	
Question #2												
Correct	61.8	58.0	ns		54.4	70.2	0.01		49.4	57.7	64.0	ns
Potential harm	38.2	42.0			45.6	29.8			50.6	42.3	36.0	
Question #3												
Correct	91.2	86.8	ns		89.2	84.5	ns		88.9	92.3	86.6	ns
No harm	8.8	11.3			9.7	13.1			8.6	7.7	12.2	
Potential harm	-	1.9			1.0	2.4			2.5	-	1.2	
Question #4												
Correct	45.6	64.6	0.01		62.6	53.6	ns		70.4	53.8	55.8	ns
No harm	48.5	33.5			35.4	41.7			25.9	42.3	41.9	
Potential harm	5.9	1.9			2.1	4.8			3.7	3.8	2.3	
Question #5												
Correct	33.8	43.4	ns		39.0	46.4	ns		38.3	30.8	44.2	ns
No harm	66.2	55.7			60.5	52.4			60.5	65.4	55.8	
Potential harm	-	0.9			0.5	1.2			1.2	3.8	-	
Question #6												
Correct	39.7	64.0	0.001		55.9	57.9	ns		61.7	53.8	56.7	ns
No harm	55.9	29.9			37.9	36.3			34.6	42.3	36.3	
Potential harm	4.4	6.2			6.2	4.8			3.7	3.8	7	
Question #7												
Correct	76.5	74.5	ns		78.5	66.7	ns		76.5	73.1	74.4	ns
No harm	22.1	20.8			17.9	28.6			29.8	23.2	21.5	
Potential harm	1.5	4.7			3.6	4.8			3.7	3.8	4.1	
Question #8												
Correct	44.1	58.3	0.04		56.4	51.2	ns		45.7	50.0	59.9	ns
No harm	55.9	41.7			43.6	48.8			54.3	50.0	40.1	
Question #9												
Correct	72.1	67.3	ns		71.1	61.9	ns		63.7	69.2	70.3	ns
No harm	22.1	20.9			18.6	27.4			22.5	26.9	19.8	
Potential harm	5.9	11.8			10.3	10.7			13.8	3.8	9.9	
Question #10												
Correct	63.2	57.1	ns		62.6	50	ns		56.8	61.5	59.3	ns
No harm	36.8	42.9			37.4	50			43.2	38.5	40.7	

ns: non-significant.

#### Medical practice setting

No significant differences were found in the frequencies of response for Questions #3, #4, #5, #6, #7, #8, #9, and #10 based on medical practice setting (outpatient clinic/office *versus* both outpatient clinic/office and hospital). However, respondents working in both settings, compared with those working only on outpatient clinic/office, had significantly higher frequencies of correct answers for Questions #1 (81.0% *versus* 66.2%, respectively; *p* = 0.01) and #2 (70.2% *versus* 54.4%, respectively; *p* = 0.01) (**Table 2**).

#### Medical practice sector

No significant differences were found in the frequencies of responses for any question based on medical practice sector (public and/or private) (**Table 2**).

## DISCUSSION

This is the first study conducted in Brazil evaluating how endocrinologists manage adrenal insufficiency in situations that can precipitate adrenal crisis, along with their practices regarding patient education. The findings of our study revealed moderate adherence to guidelines among Brazilian endocrinologists, with an overall correct response rate of 63.3%. While the highest percentage of correct answers was observed for Question #3, which concerned glucocorticoid coverage during a cesarean section (87.9%), a troubling high proportion of responses to Question #2, which addressed the self-administration of glucocorticoid via intramuscular route during situations of stress, could pose potential harm to the patient or risk of adrenal crisis (41.1%). Understanding current practices in patient, family, and caregiver education is crucial for developing effective strategies to reduce the morbidity and mortality associated with adrenal insufficiency. The present study provides valuable insights into the approaches of Brazilian endocrinologists to glucocorticoid dose adjustments during stress and their patient education/counseling practices, revealing both parallels and critical gaps compared with existing literature. These results highlight strengths and areas for improvement, particularly concerning physician education and patient guidance. Indeed, a study conducted by Kampmeyer and cols. (^11^) across ten hospitals in Germany, using a questionnaire with ten multiple-choice questions on adrenal insufficiency symptoms and treatment, administered to physicians, revealed that, considering all responses, 72.9% were correct. Furthermore, while emergency treatment was deemed essential by 83.7%, only 20 physicians (9.6%) correctly identified all situations necessitating therapy adjustment.

Data on patient adherence to glucocorticoid self-administration during stress are limited, but existing studies reveal significant challenges related to patient knowledge and skills. Harsch and cols. (^12^) found that 46% of patients were unable to manage their corticosteroid therapy during stressful events. A similar scenario was found in a German study (^13^) in which only 63% of patients felt adequately informed about stress dosing. This knowledge gap likely contributes to low self-administration rates during adrenal crises. A large international study (^7^) reported that only 12% of patients experiencing an adrenal crisis self-administered a glucocorticoid injection. This finding aligns with the results obtained from smaller studies. A UK survey (^14^) found that only 2 out of 26 patients were capable of self-administering parenteral hydrocortisone, despite established healthcare policies promoting this skill. Furthermore, a prospective, multicenter, questionnaire-based study (^15^) showed that glucocorticoid was administered by a hospital physician in most cases of adrenal crisis (55.9%), while self-injection and relative-assisted injection accounted for only 32.2% and 15.3% of the cases, respectively. Similarly, according to the findings of the study by Hahner and cols. (^16^), despite all patients (n = 37) possessing an emergency card, only 7 of them (19%) were trained in glucocorticoid self-injection. While intramuscular hydrocortisone is a cornerstone of adrenal crisis prevention (^2^), effective patient education and training for self-administration remain challenging. Our study revealed an additional concern: a substantial proportion of responses (41.1%) to Question #2 suggested potential patient harm or risk of adrenal crisis, highlighting the need for improved education not only for patients but also for assisting physicians. Promoting proficiency in glucocorticoid self-administration is important not only for reducing individual morbidity and mortality but also for decreasing healthcare system utilization and costs. Burger-Stritt and cols. (^15^) observed that patients who self-injected glucocorticoid were more likely to be treated on an outpatient basis (62%) compared with those receiving glucocorticoid from a medical professional (27%). It is important to consider the availability of medications within the Brazilian context. Injectable hydrocortisone is primarily available in hospitals, which may limit patient access to this medication for self-administration. This limited availability may influence physician recommendations, with some experienced physicians potentially advising patients to increase oral prednisone doses as an alternative strategy in emergency situations. Nevertheless, a significant advancement in supporting patient self-management is the availability of a free emergency kit containing parenteral hydrocortisone, provided by the *Associação Brasileira Addisoniana* (ABA), available at https://www.abaddison.org.br/aba-kitemerg, upon physician request. Therefore, improved physician and patient education regarding glucocorticoid self-administration during stress is essential for reducing adrenal insufficiency-related morbidity and mortality, a critical public health goal in Brazil and worldwide.

The disparity observed among endocrinologists in choosing the correct responses across the clinical scenarios underscores the need for targeted educational interventions. While questions pertaining to acute, life-threatening scenarios, such as cesarean section management (Question #3), yielded a high correct response rate (87.9%), suggesting proficiency in managing severe stressors requiring immediate intervention (^2^), questions addressing more common, albeit less acute, scenarios demonstrated lower adherence to guideline recommendations. For example, questions about management during flu-like syndromes (Questions #7 and #8) or minor procedures (*e.g.*, dental calculus removal, Question #5) revealed significant gaps in knowledge. This pattern mirrors findings by Hahner and cols. (^4^) and White & Arlt (^7^), who reported that adrenal crises frequently result from inadequate glucocorticoid dose adjustments during mild-to-moderate stress, such as gastrointestinal infections and flu-like illnesses - factors often underestimated by clinicians. This is particularly concerning given the significant risk of glucocorticoid underdosing in precipitating an adrenal crisis. Although systematic dose-response studies are lacking, and thus recommended glucocorticoid doses for adrenal crisis treatment are largely empirical, the 2016 Endocrine Society Consensus Statement (^2^) provides specific recommendations for glucocorticoid dose adjustment in various clinical scenarios, which should be the basis of clinical practice.

The relationships between physician demographics and responses to several clinical vignettes present a complex picture. While the mean age of the respondents who answered Questions #3 (cesarean section) and #6 (gastroenteritis) correctly fell between that of those who chose potentially harmful/risky responses (older group) and those who choose non-harmful/non-risky responses (younger group), a higher rate of correct answers for Questions #4 (vaginal delivery), #6, and #8 (afebrile flu) was observed among physicians with over 5 years of experience. This seemingly contradictory pattern may reflect the nuanced role of practical experience in the management of adrenal insufficiency. Specifically, older physicians tended towards more conservative recommendations regarding glucocorticoid stress dosing, which, while leading to potentially harmful/risky responses in the scenarios presented in Questions #3 and #6, resulted in the correct answer for Question #8, in which stress dosing was unnecessary. While the cross-sectional design of our study limits our ability to identify contributing factors to these observations, the overall trend underscores the critical need for ongoing medical education in adrenal insufficiency management. The lower performance of recent graduates suggests a potential gap in medical school and postgraduate training, including residency programs, regarding these specific clinical scenarios. A thorough review of medical education curricula is warranted to ensure comprehensive, evidence-based education in the management of adrenal insufficiency and the prevention of adrenal crisis.

An interesting finding emerged when comparing physician responses based on practice setting. Physicians working in both outpatient clinics and hospital environments performed significantly better on questions related to patient education (#1) and emergency glucocorticoid administration (#2). This likely reflects their broader exposure to acute care scenarios, consistent with the emphasis given by Grossman and cols. on the importance of clinical judgment in adrenal insufficiency evaluation and management (^17^). Nevertheless, Harbeck and cols. (^18^) in a cross-sectional study conducted with physicians from an Internal Medicine Department of a university hospital, found that the current knowledge of physicians regarding medical replacement strategies in adrenal insufficiency may be insufficient, depending on their level of education and experience. Even physicians with training in endocrinology demonstrated, in part, significant knowledge gaps. There may be a need for additional structured information and training on adrenal insufficiency, even in specialized hospitals.

This study has some limitations that should be acknowledged. While the pre-calculated sample size was not reached in some states, this was balanced by a larger participation in other states, resulting in the achievement of the overall target sample size. Furthermore, the study’s focus on adrenal insufficiency education may have introduced some degree of information bias. Participants, aware of the study’s objectives, may have been more likely to report behaviors they perceived as desirable or aligned with those objectives. Another limitation of this study is that the questionnaire did not explicitly differentiate between primary and secondary adrenal insufficiency, which may have introduced variability in the responses. The dissemination of the study through WhatsApp groups may have introduced some selection bias. Endocrinologists active in these groups may be more knowledgeable and up-to-date than the broader population of Brazilian endocrinologists, potentially limiting the generalizability of our findings. In short, if the participants’ low performance on certain questions raises concerns about the risk of adrenal crisis in patients, the situation in real-life settings could be even more alarming. Due to the objective and direct design of the inquiries, only prednisone was specified as the glucocorticoid, with hydrocortisone omitted based on the rationale that prednisone is the most prevalent glucocorticoid in Brazilian clinical practice due to its greater availability. For both glucocorticoids, the inquiries could have incorporated more detailed information concerning dosages across diverse clinical scenarios. Furthermore, the inclusion of family members or legal guardians in clinical education, in addition to patients, is of significant importance. Finally, while our questionnaire focused primarily on glucocorticoid administration, it is important to acknowledge that the management of adrenal crisis involves more than just corticosteroid replacement. Supportive measures, such as fluid resuscitation, electrolyte correction, and identifying and treating the precipitating cause, are critical. Furthermore, screening for serious illness is essential in the emergency setting.

While presenting limitations, the present study has some positive aspects and offers valuable insights. Participants were recruited from nearly all Brazilian states, and their distribution across regions closely mirrored the national population distribution reported by the Instituto Brasileiro de Geografia e Estatística (^19^). The Southeast region, which concentrates both population and health services, had the highest representation. Furthermore, this study is novel in its approach. To our knowledge, no prior research has used a questionnaire with clinical vignettes to assess how endocrinologists guide and manage stress in patients with adrenal insufficiency, making direct comparison with existing literature challenging. This format allowed us to pinpoint specific areas of expertise and knowledge gaps among these professionals regarding critical aspects of adrenal insufficiency care. Addressing the identified lack of awareness concerning patient education on glucocorticoid dose adjustment and self-administration during stress is essential for developing targeted continuing medical education interventions. Currently, national data on the proportion of patients receiving adequate instruction on these topics are lacking.

In conclusion, the average correct response rate was approximately 63%, indicating a moderate level of knowledge regarding patient education in adrenal insufficiency, particularly among physicians who graduated more than 5 years ago. However, the high frequency of physicians (41%) who did not provide guidance on self-administration of parenteral glucocorticoid was concerning. Identifying the reasons for this knowledge gap is essential for developing effective continuing medical education strategies.
